# Correction: Comparison of EEG-Features and Classification Methods for Motor Imagery in Patients with Disorders of Consciousness

**DOI:** 10.1371/journal.pone.0102626

**Published:** 2014-07-07

**Authors:** The PLOS ONE Staff

## Notice of Republication

This article was republished on June 19, 2014, due to the figures being ordered incorrectly. Please download the PDF again to view the corrected article. The originally published, uncorrected article and the republished, corrected article are provided here for reference.

## Supporting Information

File S1 Originally published, uncorrected article
(PDF)

File S2 Republished, corrected article
(PDF)
