# Supplementary material for: Correction: Comparison of EEG-Features and Classification Methods for Motor Imagery in Patients with Disorders of Consciousness
Source: PLoS One. 2014 Jul 7;9(7):e102626. doi: 10.1371/journal.pone.0102626 (PMC4085018; doi:10.1371/journal.pone.0102626)
Supplement: File S2 — Republished, corrected article (PDF) [file pone.0102626.s002.pdf]

OPEN ACCESS Freely available online

Comparison of EEG-Features and Classification Methods for Motor Imagery in Patients with Disorders of Consciousness

Yvonne Höller[1,2,3]*, Jürgen Bergmann[2,4], Aljoscha Thomschewski[1,3,4], Martin Kronbichler[2,4], Peter Höller[1,3], Julia S. Crone[1,2,4], Elisabeth V. Schmid[1,2], Kevin Butz[1,4], Raffaele Nardone[1,3], Eugen Trinka[1,3]

1 Department of Neurology, Christian-Doppler-Klinik, Paracelsus Medical University, Salzburg, Austria, 2 Neuroscience Institute & Center for Neurocognitive Research, Christian-Doppler-Klinik, Paracelsus Medical University, Salzburg, Austria, 3 Spinal Cord Injury and Tissue Regeneration Center, Paracelsus Medical University, Salzburg, Austria, 4 Department of Psychology & Center for Neurocognitive Research, University of Salzburg, Austria

Abstract

Current research aims at identifying voluntary brain activation in patients who are behaviorally diagnosed as being unconscious, but are able to perform commands by modulating their brain activity patterns. This involves machine learning techniques and feature extraction methods such as applied in brain computer interfaces. In this study, we try to answer the question if features/classification methods which show advantages in healthy participants are also accurate when applied to data of patients with disorders of consciousness. A sample of healthy participants (N = 22), patients in a minimally conscious state (MCS; N = 5), and with unresponsive wakefulness syndrome (UWS; N = 9) was examined with a motor imagery task which involved imagery of moving both hands and an instruction to hold both hands firm. We extracted a set of 20 features from the electroencephalogram and used linear discriminant analysis, k-nearest neighbor classification, and support vector machines (SVM) as classification methods. In healthy participants, the best classification accuracies were seen with coherences (mean = .79; range = .53−.94) and power spectra (mean = .69; range = .40−.85). The coherence patterns in healthy participants did not match the expectation of central modulated μ-rhythm. Instead, coherence involved mainly frontal regions. In healthy participants, the best classification tool was SVM. Five patients had at least one feature-classifier outcome with $p < 0.05$ (none of which were coherence or power spectra), though none remained significant after false-discovery rate correction for multiple comparisons. The present work suggests the use of coherences in patients with disorders of consciousness because they show high reliability among healthy subjects and patient groups. However, feature extraction and classification is a challenging task in unresponsive patients because there is no ground truth to validate the results.

Citation: Höller Y, Bergmann J, Thomschewski A, Kronbichler M, Höller P, et al. (2013) Comparison of EEG-Features and Classification Methods for Motor Imagery in Patients with Disorders of Consciousness. PLoS ONE 8(11): e80479. doi:10.1371/journal.pone.0080479

Editor: Esteban Andres Fridman, Weill Cornell Medical College, United States of America

Received May 7, 2013; **Accepted** October 3, 2013; **Published** November 25, 2013

Copyright: © 2013 Höller et al. This is an open-access article distributed under the terms of the Creative Commons Attribution License, which permits unrestricted use, distribution, and reproduction in any medium, provided the original author and source are credited.

Funding: This research project is being supported by the Jubil aumsfonds of the National Bank of Austria (project no. 14201).The funders had no role in study design, data collection and analysis, decision to publish, or preparation of the manuscript.

Competing Interests: The authors have declared that no competing interests exist.

* Email: y.hoeller@salk.at

Introduction

Voluntary brain activation has been extensively examined in disorders of consciousness (DOC). The goal of these endeavors is to develop a diagnostic tool to distinguish unresponsive from responsive patients if the latter are severely paralyzed and cannot react behaviorally to external stimuli. In these studies, simple instructions were presented which can be carried out by thought [1–13]. A possible command-following can be detected in the resulting brain activation with imaging methods (functional Magnetic Resonance Imaging, fMRI) or neurophysiology (Electroencephalogram, EEG). The perhaps most promising approach to apply such a diagnostic tool is based upon techniques from brain computer interface (BCI) research. Cruse et al. [3] developed a procedure, in which the patients' responses were classified with machine learning techniques. A support vector machine classified bandpass-filtered activity as recorded by central positioned channels of the EEG. Most interestingly, single patients seemed to show command following. However, the results of this research are not undisputed. Goldfine et al. [14] demonstrated that the data in this study did not meet the assumption of the statistical model, which led to artificially low p-values. By use of an appropriate permutation test the authors showed that there was no evidence for command following in any of these patients. Indeed, false positives are a major problem when applying machine learning techniques to patients with DOC. In BCI research, it is evident that the participants of the studies are performing the tests. In DOC research, we hardly know whether a patient follows the command or not. In addition, data from DOC patients differs from usual BCI-data because of the many artifact-sources such as stereotypical movements but also pathologic brain activity. These circumstances pose extraordinary demands on the data-analysis.

Cruse et al. [3] used bandpass-filtered data and a support vector machine (SVM) for classification. The healthy participant group

yielded an average classification accuracy of .68 in those cases which could be classified significantly above chance. This average did not include three subjects who showed a classification accuracy between .44 and .53. Thus, the average on all 12 participants may be around .63$\pm$. The goal of this study was to start the discussion on EEG-features for detection of voluntary brain activation in patients with DOC. Therefore, we investigated the accuracy of other markers and other classification methods in healthy participants and applied them to the data of DOC patients.

A meaningful feature/classifier yields a high accuracy among healthy participants and is reliable among healthy subjects, i.e., classifies the data of healthy participants above chance. The higher the sensitivity of a marker in healthy participants is, the higher is the sensitivity in patients with DOC. In other words, in healthy participants there should be no below-chance classification accuracy. Although a lot of BCI research concerning the optimal feature and best classifier selection has been conducted, this is seldom the case because there is a high inter-individual variability between subjects in the brain activity during motor imagery [15]. There are subjects without significant alternation of the μ-rhythm, and thus, it is possible that classification of motor-imagery related activation fails. However, there can be other relevant sources of information in the recorded EEG of these subjects, perhaps allowing to classify the data even without modulation of the μ-rhythm. To take this option into account we used a nearly full montage of EEG-electrodes instead of a small number of central electrodes, which would capture only the activity of the motor cortex. In addition, for frequency-based features, we included frequencies ranging from delta to high-beta, to capture, e.g., non μ-frequency activity related to executive functions which are involved in imaginary tasks.

In addition to a high accuracy and a low rate of non-responders, a good feature should show up with a small variance between participants, i.e., a small range and a small standard deviation (SD). Generally, these questions were and are subject of BCI research, and there are numerous studies comparing different features to each other in healthy participants. However, the examination of different features in DOC patients poses some additional demands. As stated above, false-positive results in patients are undesirable. Based on the assumption that the conducted behavioral diagnosis, carried out with the Coma-Recovery-Scale revised (CRS-R) by trained experts, which is the gold standard, is highly accurate [16], we assumed that DOC patients are not able to perform motor imagery and, thus, should not reveal above chance accuracies in the discrimination of motor imagery vs. rest trials. However, because of classification failures, for example, in unbalanced data sets, it is possible to obtain artificial high classification accuracies. This possibility and the multiple comparison problem require an adequate correction for false discoveries. We examined both, results with and without false-discovery-rate (FDR) correction since correcting the alpha level depresses the probability of Type I error but increases the probability of Type II error. We searched for features which reveal high classification accuracies in healthy participants which are significantly above chance (after FDR correction) and yield consistent results in patients. We considered results as consistent if they were significant despite correcting for multiple comparisons.

In the presented study, we recorded the EEG in healthy participants, patients in a minimally conscious state (MCS), and with unresponsive wakefulness syndrome (UWS) during imagery of moving both hands and during a rest condition (for details, see Figure 1). We extracted EEG-features to measure synchrony, complexity/entropy and to describe frequency characteristics. These features were chosen because of evidence for their value as features, for feature reduction or similar applications in BCIs or DOC (e.g. Hjorth parameters [17], brainrate [17], Wackermann features [17], power spectra [5,17], coherence [18,19], directed transfer function (DTF) [20,21], approximate entropy [22], Shannon entropy [23], Bhattacharyya distance [24]) or in other fields of EEG-research (e.g. [25]).

We compared 3 common algorithms as classification methods, discriminant analysis with a diagonal quadratic function (DADF), k-nearest neighbor classification (knn) with k = 1 and k = 3, and a support vector machine (SVM) with a linear kernel function. Previous work examined similar comparisons of classification methods and found no differences [26] or found differences which depend on the examined subject and feature [27]. There are results supporting the use of Bayesian linear discriminant analysis [28] or a stepwise linear discriminant analysis [29]. Other researchers report a slight advantage of nonlinear classification methods such as SVM over linear ones such as linear discriminant analysis [30]. There is also evidence that SVM perform much better than knn [31], but we supposed that the choice of k may influence the results.

In this study, we wanted to investigate which classification methods and features resulted in best accuracies in healthy subjects. In addition, we examined the effect of the choice of a particular feature in patients compared to healthy controls to address the question if specific features are more appropriate when investigating patients with DOC.

Results

Classification methods

Healthy participants. The results in the healthy participant group differed depending whether or not FDR was applied to the proportional chance (c_{prop}) criterion. The results of the Wilcoxon signed-rank tests comparing the different classification methods to each other in healthy participants are shown in Table 1. Without FDR-correction, it becomes evident that SVM classification (mean accuracy = 0.59) yields significantly better results than knn classification with k = 1 (mean accuracy = 0.56) and k = 3 (mean accuracy = 0.57). The reason for a high p-value in the test between DADF (mean accuracy = 0.57) and SVM may be that those features resulting in the highest classification results (coherence) did not work in DADF and thus, decreased the degrees of freedom and did not influence the test result. This problem biased also the results of the comparisons with FDR correction. However, with FDR-correction a lower number of participants yielded above chance accuracies with DADF classification in the feature-set without coherences, demonstrating that DADF yields poor results.

In summary, the differences between classification methods are rather small, but the best classification result was achieved with SVM classification.

Patient groups. There were no significant differences between classification methods in the patient groups (with p-values ranging from .5 to 1), neither with nor without FDR correction. Therefore, we chose the classification method which worked best in healthy participants for the comparison of features to each other. That is, the comparison was carried out on the SVM-results.

Features

Classification accuracies. Table 2 shows the SVM results, i.e., for each feature the average accuracies, SD, and range for all three groups (healthy subjects, MCS, and UWS). For the explanation of the features we refer to the Methods section. Note that the average accuracies of some healthy participants and

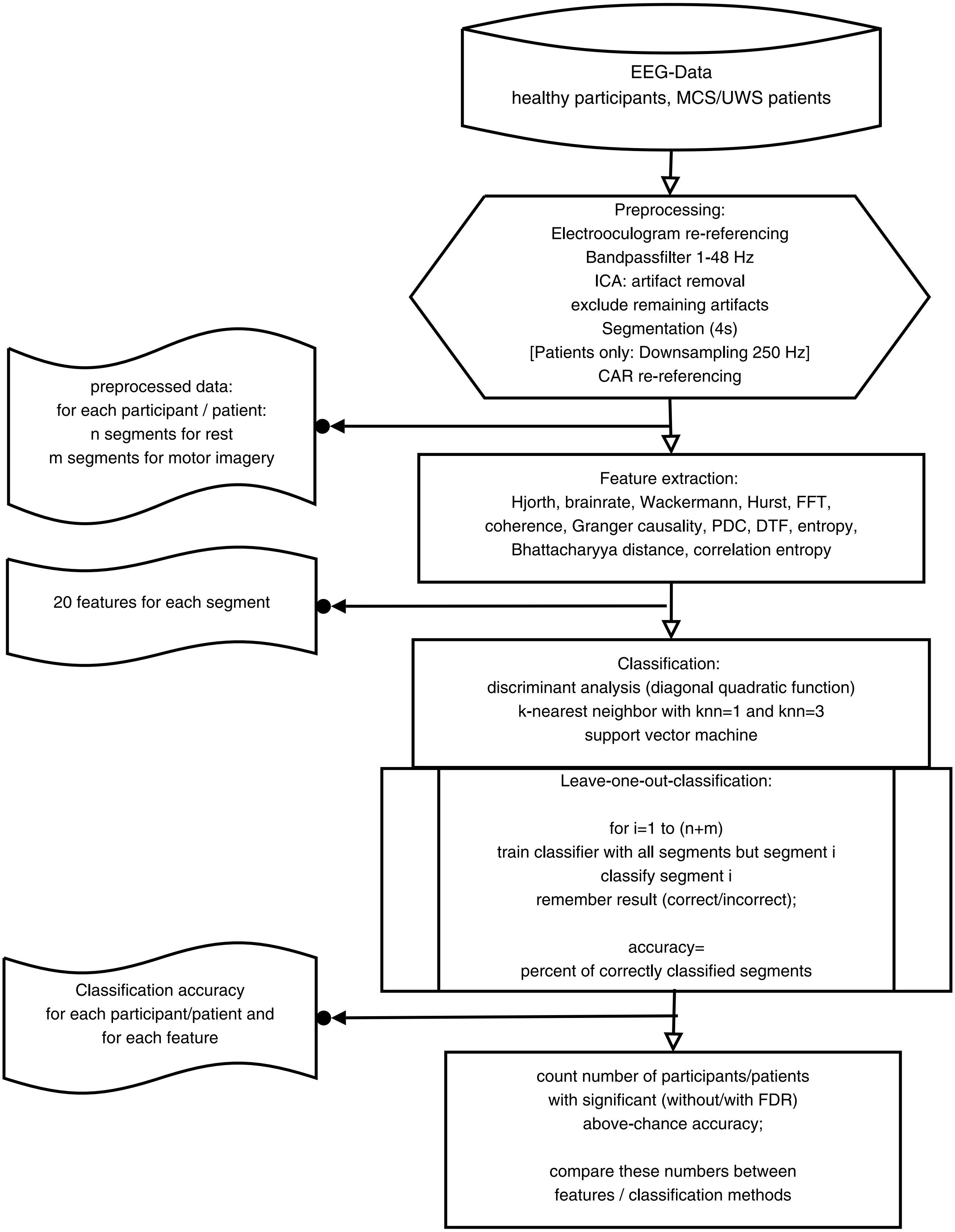

Figure 1. Procedure of data preprocessing, feature extraction, classification, and classification evaluation.
doi:10.1371/journal.pone.0080479.g001

Table 1. Comparison of classification methods.

comparison	z-value	p-value	rank
**without FDR correction**			
DADF vs. knn k = 1	−1.22	.22	51
DADF vs. knn k = 3	−0.15	.88	82
DADF vs. SVM	−1.92	.05	42
knn k = 1 vs. knn k = 3	−1.32	.19	49
knn k = 1 vs. SVM	−3.25	.001*	15
knn k = 3 vs. SVM	−2.45	.01*	21
**with FDR correction**			
DADF vs. knn k = 1	−3.87	.0001*	0
DADF vs. knn k = 3	−4.47	<.0001*	0
DADF vs. SVM	−3.98	<.0001*	0
knn k = 1 vs. knn k = 3	n.a.	.29	9
knn k = 1 vs. SVM	n.a.	.53	16
knn k = 3 vs. SVM	n.a.	.11	3.5

Results of Wilcoxon-tests comparing the number of healthy participants with above-chance accuracies.
Upper set based on above-chance numbers without FDR-correction, lower set with FDR-correction.
*significance at FDR-corrected level $p \leq .01$ n.a. not available.
doi:10.1371/journal.pone.0080479.t001

patients in certain features (e.g., Wackermann features) yielded below-chance accuracies.

Table 3 shows the average p-values and the average improvement over chance criterion (IOCC)-values for each group and each feature. Note that the p-value is also low for significant below-chance accuracy. Such a result is indicated by a negative effect size, i.e., a negative IOCC value.

Healthy participants. As can be seen in Table 2, in healthy subjects coherence yielded the highest average classification accuracy followed by FFT.

The lowest SD in the healthy participants group was found for coherence, Hurst exponent, and brainrate. However, Hurst exponent and brainrate showed very low classification accuracies in the healthy participant group.

The smallest range in the healthy participant group was found for Hjorth activity and complexity, and, again, for Hurst exponent and brainrate. Hjorth complexity had a rather small classification accuracy.

Patient groups. In patients in MCS, the highest average accuracy was found for DTF, followed by Hurst exponent and brainrate. In UWS patients, the highest average accuracy was found for brainrate.

In patients in MCS, the lowest SD was found for Tsallis knn, followed by coherence and Hjorth complexity. In UWS patients, the lowest SD was found for Hjorth activity, followed by Granger causality (Granger GW).

In patients in MCS, the significantly lowest ranges corresponded with the lowest SDs. In UWS patients, the lowest range was found for Hjorth activity, followed by partial directed coherence (PDC) and Granger GW.

Above-chance classification. The numbers of participants with above chance accuracy are shown in Table 4.

Healthy participants. The highest number of healthy participants with above chance accuracy without FDR-correction was found for coherence, followed by FFT. Both of these features yielded significantly higher numbers of above chance accuracies than the other features. Figures 2, 3, and 4 show the differences in power spectra between the two conditions in healthy participants, patients in MCS, and with UWS, respectively. Figures 5, 6 and 7 show the coherence-statistics for healthy participants, patients in MCS, and with UWS, respectively.

The number of healthy participants showing above-chance accuracy decreased considerably after FDR-correction. Only coherences still yielded a significantly higher number of above-chance accuracies compared to the other features (see Table 4, FDR-columns).

Patient groups. Most interestingly, there were no patients with above chance accuracies in power spectra and coherence. Without FDR-correction, three MCS patients had an above chance accuracy in the features Hjorth activity (patient MCS3), brainrate (patient MCS2), and DTF (patient MCS5). Among UWS patients, two patients had an above chance accuracy in the features Bhattacharyya knn (patient UWS5) and CorrEntr KDE direct (patient UWS4). Because of the occasional occurrence of above-chance accuracies in the patient groups, all of these values different from zero were significant compared to the other values.

We examined the patients with above-chance accuracies a bit closer. The above-chance accuracy in brainrate (MCS2) turned out to be a classification-error since there was only one value (brainrate = 10) for all channels and all trials. Thus, there was no difference between imagery trials and rest trials. For the other patients (MCS3, MCS5, UWS4, and UWS5), there was no obvious anomaly in the data of the respective features.

However, all of the positive results in patients were not significant after FDR-correction.

Discussion

In this study we evaluated several features and classification methods to answer the question if there are features and/or classification methods which are better suited for examining motor imagery in patients with DOC. SVM classification yielded the best results compared to knn classification in healthy participants. In the group of healthy participants we found best results for coherences and advantageous results for FFTs. Most interestingly, the coherence-results exceed classification accuracies reported previously [3]. In the following we discuss that a higher accuracy in healthy subjects is not the only reason why we suggest to use coherences with DOC-patients.

Coherences and power spectra in DOC

We found that coherences and power spectra yield high classification accuracies in healthy subjects. After FDR-correction, this advantage remains for coherences, only. Our results demonstrate that the possible value of coherences might have been underestimated so far. BCI-projects commonly use frequency analysis as revealed by FFT-power spectra [5], wavelet transform [32], or bandpass filtering [3], while studies involving coherences are rather rare. Coherences, like power spectra, use spectral information and treat the signal as stationary within the analyzed window and thus, allow to detect also pathologically delayed responses in patients [5]. The additional information in coherences over power spectra are connectivity patterns, i.e., brain regions showing synchronized frequency activity.

The use of coherences for BCI control has been shown to be a good option [18,19]. However, the pattern of coherences may differ between healthy participants and patients with severe motor disabilities [33]. The reason for distinctive patterns between patients and healthy participants was interpreted by Nam et al. [33] as reduced cortical differentiation and specialization in

Table 2. Results SVM classification.

	healthy			MCS			UWS		
**feature**	*mean*	*SD*	*range*	*mean*	*SD*	*range*	*mean*	*SD*	*range*
Hjorth activity	.65	.11	*.49–.85	.45	.15	.30–.69	.46	*.04	*.40–.51
Hjorth complexity	.57	*.10	*.39–.74	.49	*.04	*.43–.53	.47	*.07	*.35–.55
Hjorth mobility	.59	.11	.37–.80	.41	.09	.28–.49	.47	.10	.33–.60
FFT	*.69	.13	.40–.85	*.50	.11	.35–.60	.41	*.07	*.29–.49
coherence	*.78	*.09	.53–.94	.49	*.03	*.45–.55	.43	.11	.28–.63
Hurst	.53	*.09	*.36–.73	*.54	*.06	.45–.62	*.48	.08	.33–.57
brainrate	.50	*.09	*.33–.70	*.53	.10	.43–.68	*.52	.09	.37–.65
Wackermann σ	.47	.23	0–.79	.47	.12	.28–.57	.36	.16	0–.51
Wackermann ϕ	.43	.20	0–.79	.34	.16	.15–.51	.44	.10	.24–.54
Wackermann ω	.43	.22	0–.79	.35	.25	0–.59	.37	.14	.09–.57
Granger GW	.64	.15	.29–.91	.43	.09	.33–.57	.37	*.05	*.30–.48
Granger pp	*.66	.12	.41–.87	.48	.09	.38–59	.43	.08	.31–.54
PDC	.57	*.10	.30–.78	.49	.17	.23–.64	.40	*.07	*.33–.50
DTF	.60	.12	.34–.84	*.55	.08	.48–.67	.40	*.06	*.33–.51
approximate entropy	.61	.11	.43–.83	.48	.10	.38–.63	.48	.09	.33–.62
Renyi spacingV	*.65	.12	*.47–.85	.41	*.06	*.32–.46	*.51	*.06	*.43–.62
Tsallis knn	.63	.12	.45–.85	.46	*.03	*.43–.49	*.48	.10	.31–.60
Shannon spacingV	*.65	.12	*.47–.85	.41	*.06	*.32–.46	*.50	*.07	*.43–.62
Bhattacharyya knn	.60	.11	.40–.80	.42	*.06	*.36–.51	*.50	.12	.33–.72
CorrEntr KDE direct	*.65	.13	.28–.87	.46	*.06	*.38–.51	*.51	.14	.30–.67

*significantly better than other features of same column (FDR $p < 0.0242$).
doi:10.1371/journal.pone.0080479.t002

patients, reflected by a compensatory recruitment of more cortical regions. This difference may lead to lower performance of a BCI-system and thus, decrease the classification accuracy in patients. Against this background, one could argue that coherences are rather unsuited for use in DOC-patients because they may underestimate a patient's ability to perform motor imagery. However, the classification accuracy of features such as power spectra and coherence possibly depend also on the electrode positions used for classification [34]. Krusienski et al. [34] reported that FFT and coherence yield comparable classification accuracies in healthy participants with central electrode positions covering the motor cortex. Nevertheless, the authors state that it is very likely that electrodes positioned over all brain regions could yield different results (i.e., advantages for coherences or phase locking values). Such a montage could detect task-related activity which increases classification accuracy but does not stem from the motor cortex. For example, one could expect a strong activation of frontal regions due to executive functions involved in task performance. This is what we found in healthy participants, i.e., significant differences between rest and imagery coherences between frontal and other brain regions. This alternative source of brain activity could be relevant in patient groups. For example, patients with spinal cord injury suffer from deafferentiation and thereby negative neuroplasticity in the sensorimotor cortex. This may explain the rather low classification accuracies in BCI-systems in tetraplegic patients [35].

Coherences could yield the possibility to recognize motor-imagery related activation even without typical activation of the motor cortex. Indeed, our approach of using a full montage revealed that healthy subjects show frontal connectivity patterns in coherences, suggesting that the high classification accuracy in this group stems not only from central activations. Thus, the way we applied coherences should be able to detect voluntary brain activation in patients with DOC even if they suffer from negative neuroplasticity in the motor cortex. A similar approach could be achieved also by features such as Granger causality (Granger GW). However, Granger causality requires a fine tuning of the lag length and produces a longer data vector, possibly causing a small-sample-size problem.

Another advantage of coherences is that they yield small SDs and rather small ranges, which makes them better suited to distinguish participants which do and do not perform motor imagery. In fact, after FDR-correction only 2 healthy participants yielded below-chance accuracy. There were also other features with small ranges or small SDs but only coherences had a small variance combined with high accuracy in the healthy participants group.

False positives

Without FDR-correction, there were some patients showing above chance accuracies in some of the features. These occurrences in the patient groups did not exceed the FDR-corrected threshold. Most interestingly, features which yielded above-chance accuracies in patients did not match with those features showing the highest classification accuracies in the healthy participant group. If the above chance accuracies in patients would have occurred in those features which yielded best results in the healthy participant group the results would have been more thrustworthy. Indeed, the brainrate-results turned out to be a severe classification error.

Only recently Goldfine et al. [14] found that noise in the data [3] may lead to failure of the classifier. Most importantly, several

Table 3. Average p-values and effect sizes (IOCCM) for comparison of achieved accuracy to chance level in the SVM classification.

	healthy		MCS		UWS	
**feature**	p	IOCCM	p	IOCCM	p	IOCCM
Hjorth activity	.14	.30	.14	−.10	.32	−.08
Hjorth complexity	.18	.14	.35	−.01	.29	−.07
Hjorth mobility	.20	.17	.23	−.19	.22	−.06
FFT Hz	.09	.37	.21	−.01	.21	−.18
coherence	.02	.56	.35	−.03	.24	−.15
Hurst	.24	.06	.27	.09	.29	−.03
brainrate	.27	−0	.25	.05	.27	.03
Wackermann σ	.14	−.06	.25	−.06	.19	−.28
Wackermann ϕ	.24	−.14	.16	−.33	.26	−.11
Wackermann ω	.20	−.14	.19	−.30	.13	−.25
Granger GW	.11	.27	.19	−.15	.16	−.22
Granger pp	.12	.32	.26	−.04	.21	−.14
PDC	.20	.13	.15	−.02	.18	−.20
DTF	.13	.20	.29	.10	.16	−.21
approximate entropy	.20	.21	.24	−.05	.26	−.04
Renyi spacingV	.15	.30	.21	−.18	.31	.01
Tsallis knn	.16	.26	.34	−.08	.23	−.03
Shannon spacingV	.15	.30	.23	−.17	.30	.01
Bhattacharyya knn	.17	.19	.21	−.16	.22	0
CorrEntr KDE direct	.10	.30	.31	−.08	.14	.02

doi:10.1371/journal.pone.0080479.t003

Table 4. Numbers of above-chance accuracies in the SVM classification.

	healthy		MCS		UWS	
**feature**	C_{prop}	FDR	C_{prop}	FDR	C_{prop}	FDR
Hjorth activity	11	2	*1	0	0	0
Hjorth complexity	5	1	0	0	0	0
Hjorth mobility	6	2	0	0	0	0
FFT Hz	*16	2	0	0	0	0
coherence	*21	*20	0	0	0	0
Hurst	3	1	0	0	0	0
brainrate	1	1	*1	0	0	0
Wackermann σ	4	1	0	0	0	0
Wackermann ϕ	1	1	0	0	0	0
Wackermann ω	2	1	0	0	0	0
Granger GW	12	1	0	0	0	0
Granger pp	11	2	0	0	0	0
PDC	3	1	0	0	0	0
DTF	6	1	*1	0	0	0
approximate entropy	5	2	0	0	0	0
Renyi spacingV	11	1	0	0	0	0
Tsallis knn	9	1	0	0	0	0
Shannon spacingV	11	1	0	0	0	0
Bhattacharyya knn	9	0	0	0	*1	0
CorrEntr KDE direct	10	1	0	0	*1	0

numbers of participants significantly above C_{prop} for each group; numbers without and with FDR-correction.
*significantly higher compared to other features of same column with FDR-corrected level of significance $p<0.0069$.
doi:10.1371/journal.pone.0080479.t004

above-chance accuracies in healthy participants as well as in patients were not significant anymore after FDR-correction. As Goldfine et al. [14] point out, based on the statistical criterion of $p<.05$, a classifier could yield positive results in 5% of the tests even if these datasets were random. In the present data, in 14 patients and 20 features there were 5 above-chance accuracies before an FDR-correction was applied. Based on the assumption that the diagnosis with the CRS-R is highly accurate [16], we considered above-chance accuracies as false positives if they did not reach significance at the FDR-corrected level. Still, the CRS-R is the gold standard, but there is no ground truth to truly ascertain the results in studies involving DOC. Therefore, we need features which are robust against artifacts and classification errors.

Perhaps just because of the possibility of false positives in other features we see the clear advantage of coherences. There were no above-chance accuracies with or without FDR correction in patients despite a high number of above-chance accuracies was found in healthy subjects. We consider it a main advantage of the coherences that they seem to be robust against erroneous classifications.

However, examining the healthy participant group showed that interpreting coherence-patterns is not straightforward. Analysis of motor-imagery-related EEG data is usually done on central positions, only, because they represent the central μ-rhythm. Thus, we expected to find significant coherences involving central positions in healthy participants. In contrast, there are more healthy participants showing significant differences in the coherences between frontal and other (frontal, central, parietal) regions than participants showing significant differences between central regions. With respect to power spectra, the difference between motor-imagery and rest is obvious above central, parietal, and occipital regions in healthy participants, but with a high SD in the range of the μ-rhythm. This inter-individual difference in activation patterns is well in line with previous research involving simple stimuli such as an oddball [36,37], subject's own name [38,39], but also complex tasks such as music [40], and specifically motor imagery [15,41], in which healthy participants show contradictory patterns of activation. As a consequence, visually exploring the pattern of coherences cannot specify whether a patient was performing the task or not. This becomes obvious when comparing the coherence-distribution in the patient groups to the distribution in the group of healthy participants. The attempt to compare power spectra between patients and healthy participants seems to be more useful. However, power spectra achieved a low number of above-chance classification in healthy participants after FDR-correction. Therefore, classification results in power spectra may underestimate a patients' task performance.

Finally, we want to mention that we cannot rule out that patients without below-chance accuracies in coherences (as in any other feature) are conscious. First, it is possible that patients were actually performing the task and the coherence feature failed to classify them correctly as reported previously for severely motor disabled patients [33]. The statistical procedure could have failed to reveal the motor imagery related brain activation or the patients are simply not be able or willing to follow the task instructions. Patients with dementia, for example, are surely conscious but at a certain stage of the progressive disease they may have difficulties in performing even such a simple task.

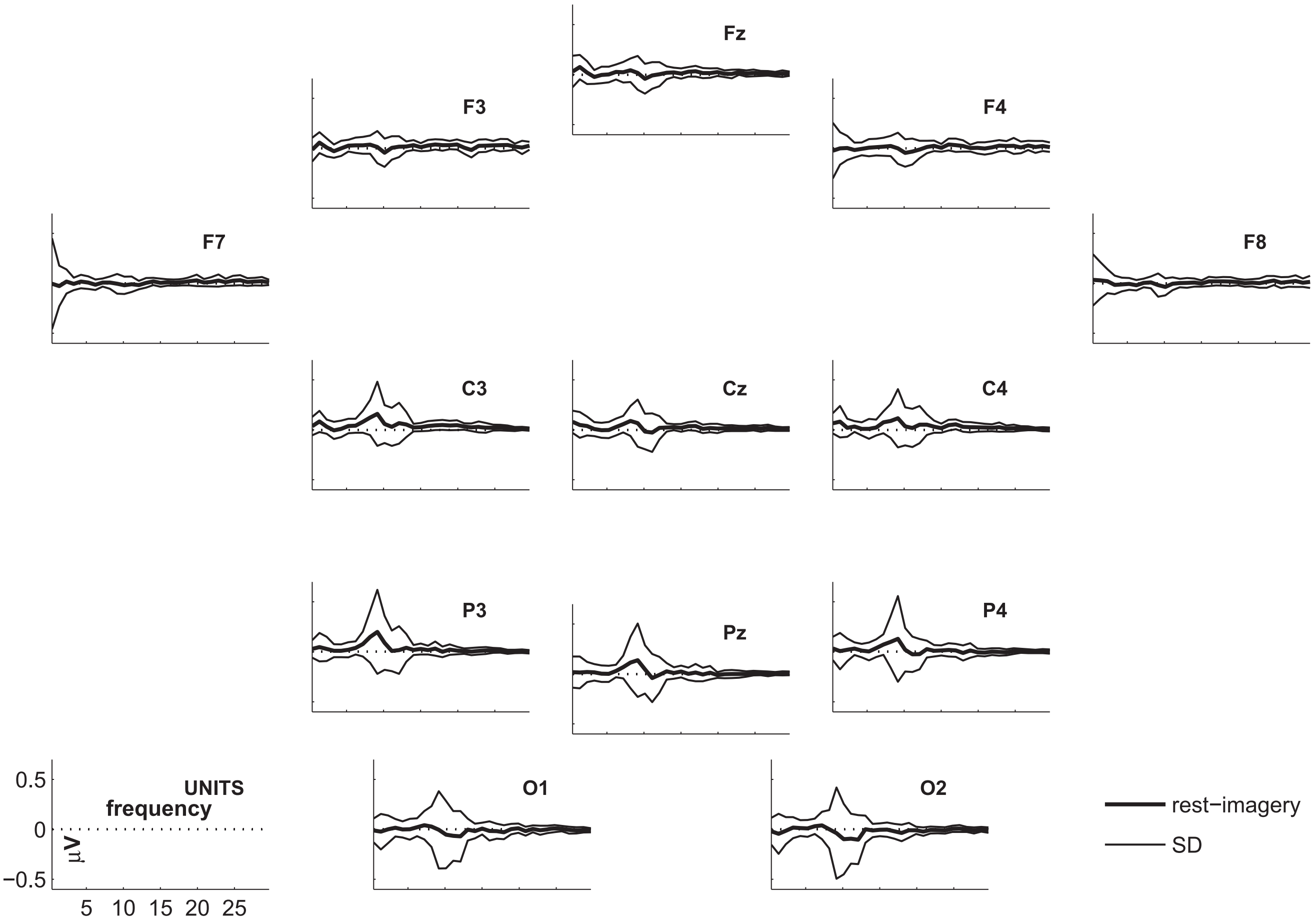

Figure 2. Differences in power spectra (rest-imagery) in healthy participants. Thick line indicates the mean of the sample, the thin lines indicate the standard deviation.
doi:10.1371/journal.pone.0080479.g002

Classification problems: Overfitting

We have found many accuracies smaller than .50 in the patient groups and some in the healthy participant group. It is very likely that the below-chance values are due to overfitting of the data. It is known that the risk for overfitting the system to a particular class rises when the number of samples used for the different classes is not well balanced. That is, the classifier will assign images more likely to the class which contains the most samples within the training set. In addition, overfitting is especially a problem with high dimensional data, i.e., long feature vectors. In EEG-data, this problem of long feature vectors is very common and there exist approaches to reduce e.g. the number of frequencies [42]. Finally, overfitting is also a problem in noisy data, because random variability in the data leads to an inaccurate model. In the worst case, overfitting leads to zero classification accuracy. This may happen in the case that both classes (motor imagery and rest) have equal numbers of trials. Let this number be N. With the leave-one-out cross-validation one trial x of class A is classified and all of the other trials of class A and B serve as training data. The training data includes N trials of class B and N-1 trials of class A. Because of overfitting, the classifier assigns the wrong class B to the trial x. As such, each trial is assigned to the wrong class and the classification accuracy is 0. In a similar way overfitting could lead to above-chance accuracy if there are strongly unbalanced data-sets.

The main reason for overfitting in DOC patients and, thus, below-chance accuracies, may be that the data contains no task-related information. For the classifier, non-task-related information is equivalent to noise. In fact, if a patient does not perform motor imagery there is no model to build for distinguishing the motor-imagery trials from rest trials.

We believe that coherences are robust against overfitting. In healthy participants, coherences were the only feature with no subjects with below-chance accuracy. The lowest accuracy for coherence among healthy participants was .53 while in all other features the lowest value was below .5. This is also reflected by the fact that coherences had a very small standard deviation and a rather small range both in patients and healthy participants. The accuracies do not vary extremely between participants. This makes the results more reliable and leads to the assumption that a coherence value is better predictable than, e.g., a Wackermann feature, which yielded many below-chance accuracies and a high between-subject variability.

Choosing SVM as a classifier for EEG-data in DOC

We found no significant difference between classifiers in patients. Therefore, the deciding factor for choosing a classifier remains the classification accuracy in healthy participants. But also in healthy participants the differences between classifiers were rather small.

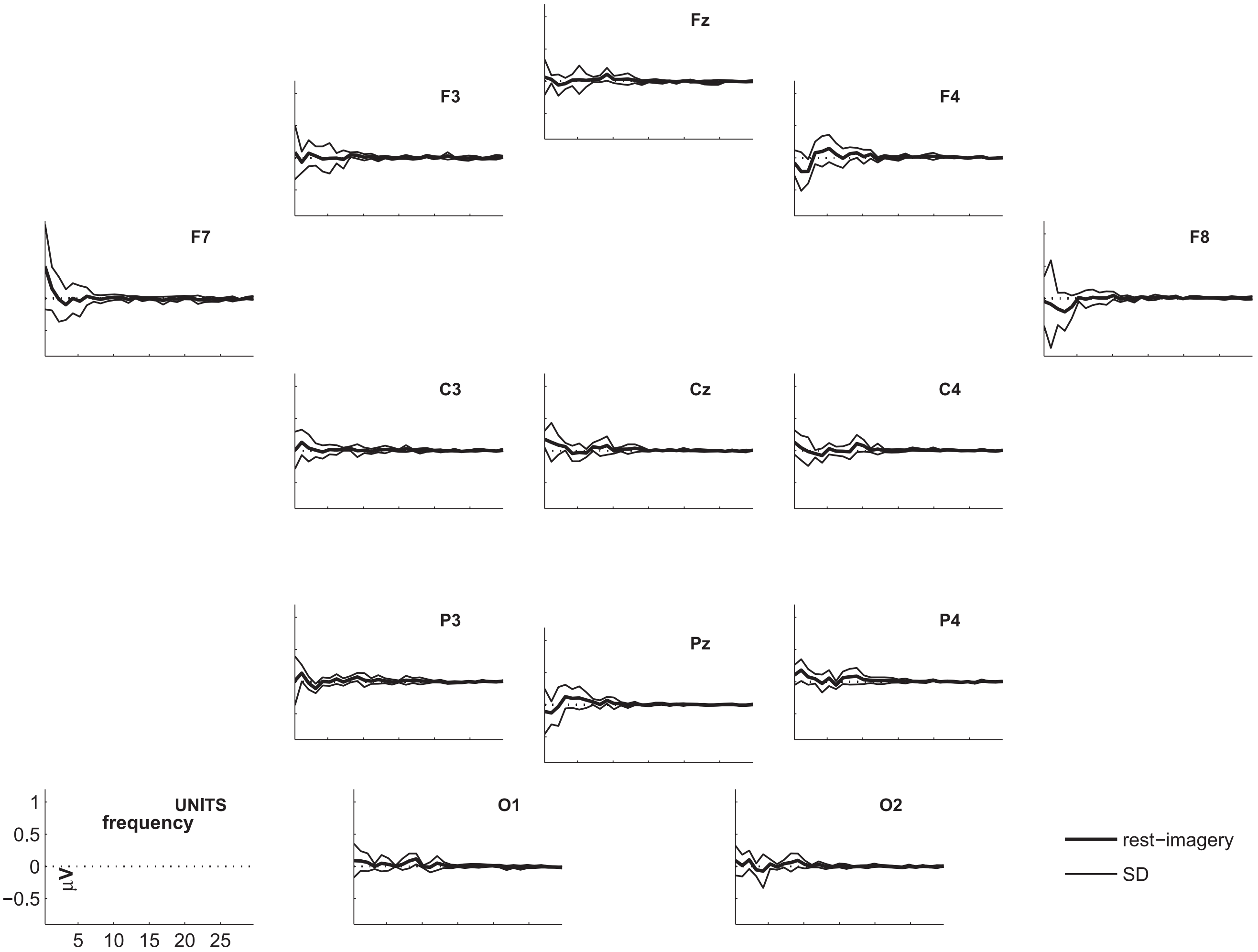

Figure 3. Differences in power spectra (rest-imagery) in patients in MCS. Thick line indicates the mean of the sample, the thin lines indicate the standard deviation.
doi:10.1371/journal.pone.0080479.g003

SVM yielded best results for healthy participants in our study, which is not surprising given the results of previous research [31,43]. SVM has been used for synchronous brain computer interfaces [32] and for classifying motor imagery in patients with DOC [3]. The main drawback of SVM is that it is slower than other classification methods. However, for the diagnosis of DOC this is not critical because data are usually analyzed off-line. If used for BCI in patients with DOC or SCI, SVM can still be fast enough to reach real-time performance [32]. Nevertheless, the differences of the classification accuracies were rather small and the difference between SVM and DADF were not significant because of a smaller number of degrees of freedom (despite the average accuracy of DADF was the same as for knn $k = 3$, which was boarder-significantly different from SVM). These results are well in line with previous research, reporting only a slightly better result for SVM than linear discriminant analysis [30]. This suggests that the differences between the classifiers are not very wide and comparing SVM with other classification methods, e.g., a Bayesian classifier may yield no difference between classification results [44]. It is important to note that the kernel of the SVM plays an important role. We used a linear kernel function, but a non-linear kernel function allows a more flexible decision boundary in the data space [32] and also a Gaussian kernel function was shown to be advantageous [44]. However, when applying a non-linear kernel function we found that the classifier did not converge to a solution for each participant's data in certain features. Thus, when choosing a classifier and tuning the classifier's parameters there is a trade-off between best classification results and reliability of the algorithm to converge.

Conclusion

In this study we compared the use of different features and different classifiers in healthy participants and patients with DOC. SVM classification yielded the best results in healthy participants among the options we tested. Classification accuracies of coherences were found to be better than those of all other features.

In DOC patients, though, we recommend to examine coherences over the whole scalp since they identify a more distributed network activity reflecting the cognitive processes involved in motor imagery. This advantage could be crucial when examining severely-disabled patients since the individual response patterns can differ drastically from those of healthy people because of neuroplastic changes. Without FDR-correction we found above-chance accuracies for patients in other features. At least one of these occurrences was identified as failure of the

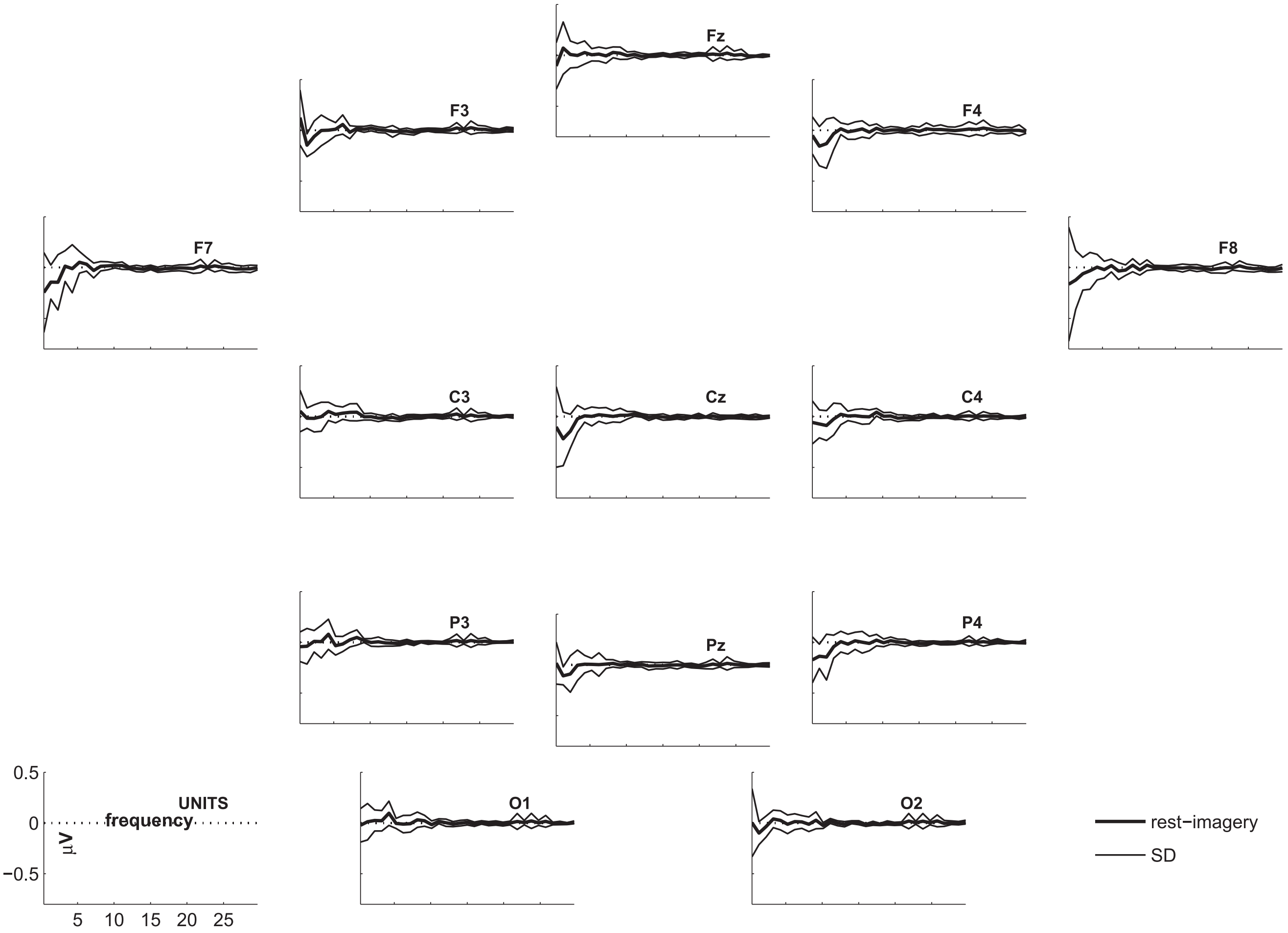

Figure 4. Differences in power spectra (rest-imagery) in patients with UWS. Thick line indicates the mean of the sample, the thin lines indicate the standard deviation.
doi:10.1371/journal.pone.0080479.g004

classification method. We suggest that coherences are more robust against artifactual data. This claim is also supported by a small variability of accuracies in coherences, suggesting a good predictability of the classification values.

Future studies should examine if classification accuracies of coherences calculated on a full montage are affected by neuroplastic changes in severely motor disabled patients.

Methods

Ethics

The study was approved by the local Ethics Committee (Ethics Commission Salzburg/Ethikkommission Land Salzburg; number 415-E/952) and was designed according to the Declaration of Helsinki. Written informed consent was obtained from all control subjects and from the families or guardianship of all patients.

Subjects

Over the course of 2 years (2010–2012), 16 patients were assessed at the Christian-Doppler-Clinic in Salzburg (Austria). Inclusion criteria for patients were a diagnosis of UWS ($n = 10$) or MCS ($n = 6$) based on neuropsychological assessment of trained experts with the CRS-R [45]. Due to artifacts resulting from stereotypical movement patterns, the data of 2 subjects (1 UWS; 1 MCS) were excluded. The remaining sample of 9 UWS patients and 5 MCS patients is described in Table 5.

Cd, code: diagnosis and patient number; WHIM, value on Wessex Head Injury Matrix; CRS-R, value on Coma Recovery Scale -Revised; Dur, duration in months of the disorder at the time of assessment since onset; N.A., not available; H, haemorrhage.A sample of 22 high school graduated subjects (age: 20–26 years; mean = 22.86 years; SD = 1.81; 6 male) was recruited for the healthy subject group. None of the participants reported any history of neurological or psychiatric diseases, nor were they receiving any psychoactive medication. Healthy subjects were remunerated for their expenditure of time. The data of the healthy subject group was analyzed and published recently [15].

Experiment

The experiment consisted of three conditions. There were 24 trials for each condition. In the imagery-condition the participants were asked to imagine to open and close both hands. The resting condition consisted of no movement and no imagery but was preceded by the instruction to hold both hands firm. With this special ''resting task'' we controlled for speech-related activations, which are common in patients with DOC and do not reflect consciousness [46,47]. To ensure that the participants performed the task in the movement- and the imagery-condition during a

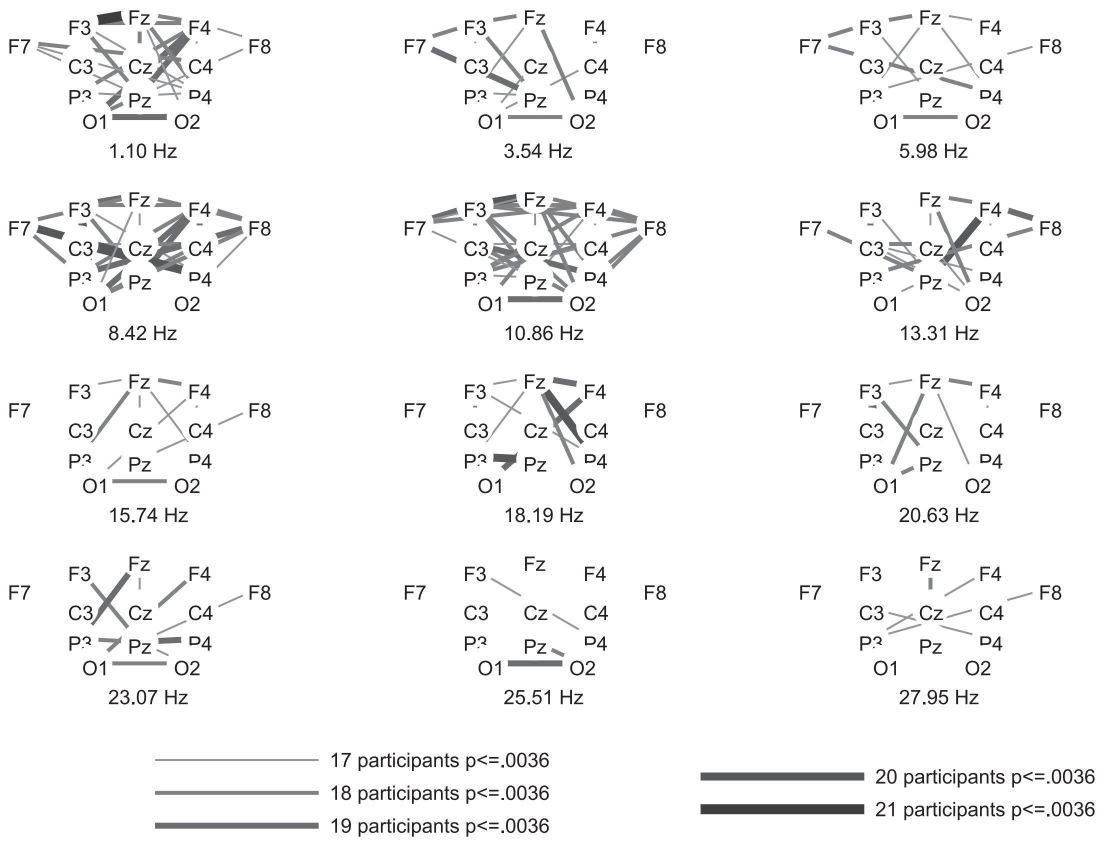

Figure 5. Numbers of healthy participants with significant (FDR-corrected) coherences.
doi:10.1371/journal.pone.0080479.g005

certain period of time, they were instructed to perform the task while hearing a tone sequence of 2 tones. Both tones were alternatingly recurring once per second. Thus, the participants imagined to open and close their hands once per second. The instructions had durations of 6, 6.5, and 9.5 sec for the rest, move, and imagery conditions, respectively. After each instruction, there was an interval of 5 sec during which the participants were expected to follow the instruction (i.e., to move in the movement-condition, to imagine a movement in the imagery-condition, and to hold the hands firm in the rest-condition). To avoid expectation effects, conditions were ordered pseudo-randomly. Instructions were presented verbally and binaurally through earphones using Presentation software (Neurobehavioral Systems, version 12). The auditory material was recorded and processed with Audacity (version 1.2.6). The instructions were normalized to an equal sound level. Healthy participants were asked to look straight ahead during the experiment. Patients were awake during the experiment with eyes open. The presented data are based on these two conditions while the third condition, the movement condition, was excluded from analysis because of limited information content in the patient groups.

Data registration

EEG-Data was recorded using a BrainCap with a 10–20 system and a BrainAmp (Brain Products GmbH, Germany) 16-bit ADC amplifier. The sampling rate was 250 Hz for the healthy participants and 1000 Hz for the patients. Of the 32 recorded channels, 2 were used to monitor the left and right horizontal electrooculogram. One was used to monitor lower-site vertical electrooculogram. Two were positioned at the mastoids for re-referencing purposes to remove the bias of the original reference, which was placed at Fcz. The other electrodes were Fp1, Fp2, F3, F4, C3, C4, P3, P4, O1, O2, F7, F8, T7, T8, P7, P8, Fz, Cz, Pz, FC1, FC2, CP1, CP2, FC5, FC6, CP5, and CP6. Data analysis was conducted for data collected from the electrodes F3, F4, C3, C4, P3, P4, O1, O2, F7, F8, Fz, Cz, Pz, FC1, FC2, CP1, CP2, FC5, FC6, CP5, and CP6. Impedances were kept below 10 kΩ.

Data preparation

An overview of this and the following steps is given in the flowchart in Figure 1. Data were pre-processed with Brain Vision Analyzer (Version 1.05.0005, Brain Products GmbH). In order to re-reference all channels, a new reference was built by averaging the signal of mastoid electrodes. To obtain a bipolar vertical electrooculogram, the average of Fp1 and Fp2 was used as a reference for the lower-site vertical electrooculogram. Butterworth Zero Phase Filters from 1 to 48 Hz (time constant 0.1592s, 48dB/oct) were applied to reduce noise.

Independent component analysis (ICA) was applied to detect and remove ocular, muscular, and cardiac artifactual sources [48–50]. The ICA was calculated on the entire dataset, on all channels, including the prepared electrooculographic channels. An experienced researcher identified the components containing ocular, cardiac, or muscle artifacts by visual inspection. These components were removed by performing the corresponding ICA back-transformation.

An automatic data inspection was carried out in order to exclude remaining artifacts. Maximal allowed voltage step per

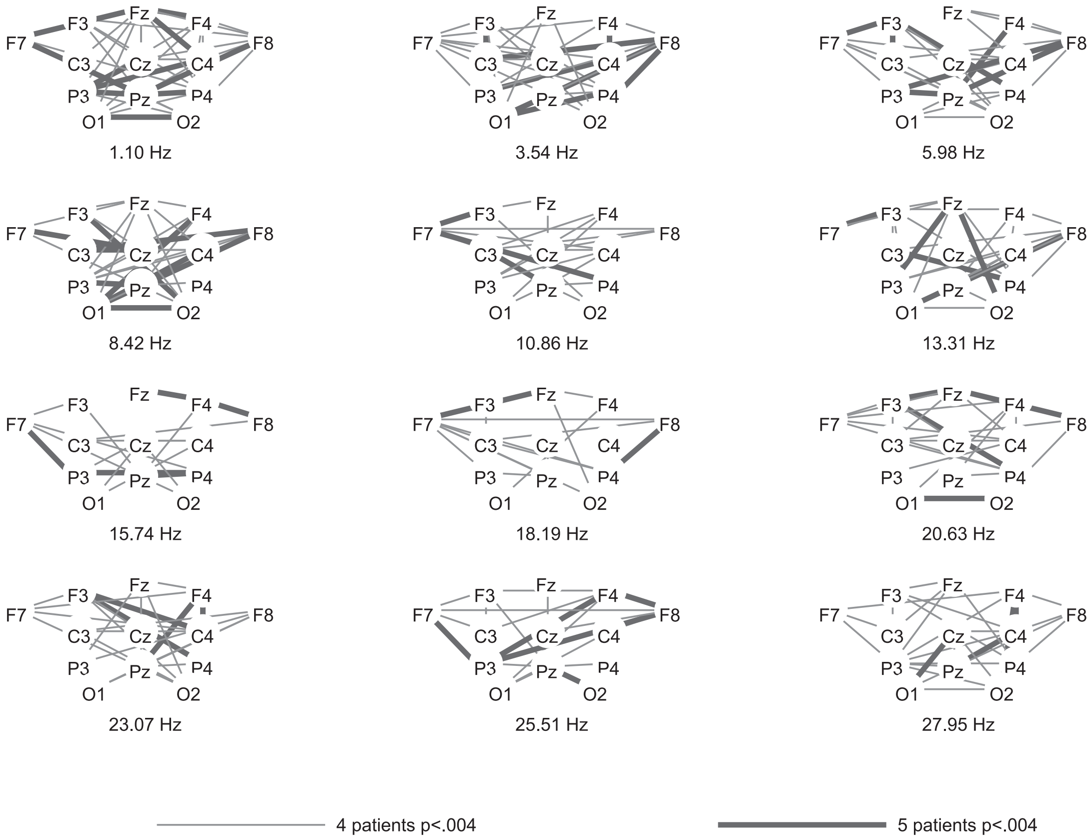

Figure 6. Numbers of patients in MCS with significant (FDR-corrected) coherences.
doi:10.1371/journal.pone.0080479.g006

sampling point was 50 μV (exceeding values were excluded with a surrounding of $\pm$100ms); maximal allowed absolute difference on an interval of 200ms was 200 μV and lowest allowed absolute difference on an interval of 100ms was 0.5 μV (exceeding values were excluded with a surrounding of $\pm$500ms).

To extract features of individual trials, data was segmented into 4 sec epochs for each trial. These segments started at the end of the acoustic instruction and ended 1 sec before the next instruction to avoid expectancy effects. The preprocessed segments were exported into a generic data format and imported to Matlab® (The Mathworks). These segments were first spatially filtered to common average reference [51].

Patient's data was first downsampled to 250Hz to have the same sampling frequency as data collected from healthy participants.

Feature extraction

In the following, we list the features and refer to the software we used to compute them.

Hjorth parameters. The BioSig implementation [52] of the Hjorth features as proposed by Hjorth [53,54] was used. Hjorth parameters include activity, mobility, and complexity. Each of these features was evaluated in stationary mode separately for each channel to the whole segment (4 sec). Thus, the result was a feature per channel and, as a whole, a feature vector. Classification was carried out separately for the three Hjorth feature vectors.

Brainrate. The BioSig implementation [52] of the brainrate [55] was used. The brainrate was calculated in stationary mode for each channel over the whole segment.

Wackermann. The BioSig toolbox [52] provides a function to calculate the global field strength σ, the global frequency ϕ, and a measure of spatial complexity ω [56,57]. Each of these features was evaluated in stationary mode separately for each channel over the whole segment. Classification was carried out separately for the three Wackermann feature vectors.

Hurst exponent. The BioSig toolbox [52] includes a function to estimate the Hurst parameter via the rescaled range [58]. The Hurst exponent, also known as index of long-range dependence, was calculated for each channel over the whole segment.

FFT. The FFT was calculated with the matlab function fft.m. To reduce the length of the feature vector (124 frequency steps between 1 and 30Hz for each channel), we calculated the average value at 2.44Hz frequency steps between 1 and 28Hz (12 values for each channel).

Coherence. Coherence was calculated with the matlab function mscohere.m. To reduce the length of the feature vector (124 frequency steps between 1 and 30Hz for each channel $\times$ channel combination) we calculated the average value at 2.44Hz frequency steps between 1 and 28Hz (12 values for each channel $\times$ channel combination).

Granger causality. Spectral granger causality [59,60] was calculated with the function cca_pwcausal.m in Seth's toolbox [61] which was applied to each single segment. This function returns the log-ratio of granger-causalities for each frequency step (GW, i.e., a matrix with channels in rows and columns for each frequency step), analogously values of coherence, and the power spectrum for each channel (pp). We used 2.44 Hz frequency steps between 1 and 28 Hz. Note that the information of these

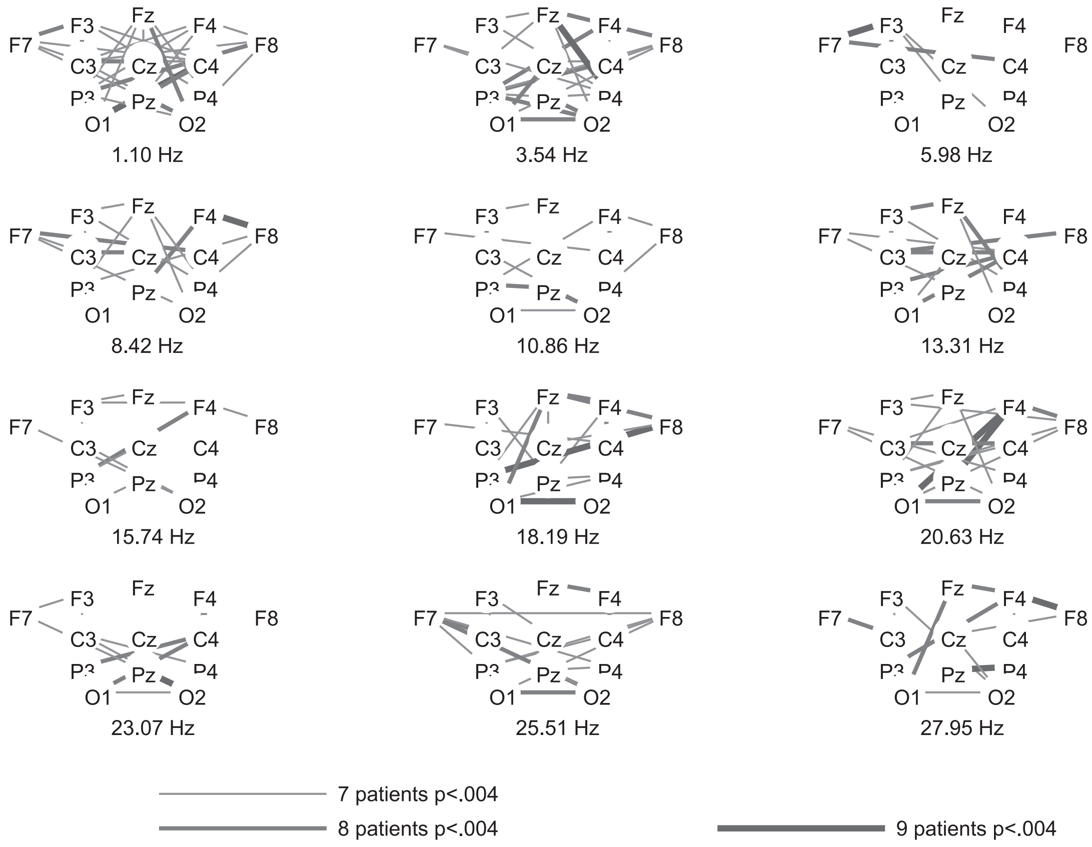

Figure 7. Numbers of patients with UWS with significant (FDR-corrected) coherences.
doi:10.1371/journal.pone.0080479.g007

coherence values overlaps with the information in the Coherence-feature vectors as calculated with the function mscohere.m. Therefore, only GW and pp were used for classification (each of them separately).

PDC and DTF. The PDC [62] and DTF [63] were calculated with functions provided by Omidvarnia [64] including also functions from the BioSig toolbox [52] and the arfit toolbox [65,66] with 12 2.44 Hz frequency steps between 1 and 28 Hz. For computing the time-varying PDC and DTF measures we used the optimum model order as estimated with the arfit toolbox between 1 and 20 for each trial.

Entropy. A large set of entropy measure calculations was first examined. Shannon entropy was calculated with the matlab function wentropy, approximate entropy was calculated with the function ApEn provided by Kijoon Lee in Matlab central file exchange with a tolerance of $r = 2 * SD$. All of the other entropy features (again Shannon entropy but by knn estimation, with multivariate Edgeworth expansion, locally linear regression, approximate slope of the inverse distribution function with and without piecewise constant or linear correction as well as with and without bias correction; Rényi entropy estimated by knn, weighted knn, empiric entropy estimator of order m, approximate slope of the inverse distribution function, and continuously differentiable sample spacing (CDSS); Tsallis entropy estimated with k-nearest neighbors) were calculated with Szabó's Octave/Matlab Toolbox for Information Theoretical Estimators [67,68]. All of these entropies were then classified in all groups and we chose the Shannon entropy and the Rényi entropy with highest classification accuracies in the healthy participant group since the values in patient groups did not differ systematically. The highest accuracies were reached with the estimation with approximate slope of the inverse distribution (spacingV). Thus, we used 4 entropy measures: Shannon and Rényi entropy estimated by spacingV, Tsallis entropy estimated with k-nearest neighbors, and approximate entropy.

Bhattacharyya distance. Bhattacharyya distance to measure the divergence, i.e. the distance between to probability densities, was calculated with the with k-nearest neighbors implementation of Szabó's Octave/Matlab Toolbox for Information Theoretical Estimators [67,68].

Correlation Entropy. Correlation entropy (CorrEntr KDE direct), an association measure, was calculated with Szabó's Octave/Matlab Toolbox for Information Theoretical Estimators [67,68].

Classification

Trials of each participant were classified with the leave-one-out cross-validation. Each trial was used once as validation data with the remaining trials as the training data. This was repeated such that each trial was used once as the validation data. Then, we calculated the percent of correctly classified trials. This proportion is called hereafter the classification accuracy.

For classification we used three algorithms (MATLAB-functions):

discriminant analysis with a diagonal quadratic function (DADF). Each trial was classified by a specified discriminant function. In the present case, this function was "diagonal quadratic". In this function multivariate normal densities are fit

Table 5. Summary of patients.

Diagn	WHIM	CRS-R	Sex	Age	Dur	Etiology
MCS-1	12	8	m	40	62	traumatic brain injury
MCS-2	15	9	m	52	4	subarachnoidal + intracerebral H
MCS-3	15	11	w	71	12	subarachnoidal H
MCS-4	10	8	w	56	20	subarachnoidal H
MCS-5	13	9	w	65	7	intracerebral H
UWS-1	6	6	w	38	18	hypoxic encephalopathy
UWS-2	1	1	m	55	2	cardiopulmonary resuscitation
UWS-3	2	4	w	32	30	basilarthrombosis
UWS-4	4	3	m	73	2	traumatic brain injury
UWS-5	5	4	m	60	2	traumatic brain injury
UWS-6	4	6	m	47	119	cardiopulmonary resuscitation
UWS-7	3	3	m	61	2	thalamic H
UWS-8	3	7	w	36	14	status epilepticus
UWS-9	5	5	m	31	2	traumatic brain injury

doi:10.1371/journal.pone.0080479.t005

with a diagonal covariance matrix estimate (naive Bayes classifiers) stratified by group.

k-nearest neighbor classification (knn). Each trial was classified by knn classification with Euclidean distance metric. We applied the knn-classifier twice, with $k=1$ and with $k=3$. These two values were chosen because with $k=1$ we produced the special case of the nearest neighbor algorithm and with $k=3$ we chose an odd number, which avoids tied votes in binary classification problems and this value for k is still small so that the boundaries between classes can be quite distinct.

support vector machine classifier (SVM). Classification with SVM technique was used with a linear kernel function.

Above-chance classification

Thus, we got a classification accuracy for each participant. Based on the individuals' classification accuracies, we estimated the number of healthy participants, UWS-patients, and MCS-patients who showed above chance accuracies, separately for the 4 classification methods and for each of the 20 features. We used a proportional chance criteria and an adequate measure of significance and effect size, as described in [69], for assessing if the features' accuracies reflected task performance, that is, if they classified motor imagery and rest-trials with an accuracy that is significantly above chance. The proportional chance criteria HC_{prop} is calculated by summing the squared proportion that each number of trials represents in the whole trial set:

$$HC_{prop} = (0.5 * N_r + 0.5 * N_i) \tag{1}$$

where N_r is the number of resting-trials and N_i is the number of motor imagery trials. We required that the accuracy should be significantly better than the chance criteria [69]. That is, an accuracy is considered as reflecting task performance if the improvement over chance criterion was significant as assessed by a z-statistic:.

$$z = \frac{H_0 - HC}{\sqrt{HC(N-HC)/N}} \tag{2}$$

where H_0 is the number of correctly classified trials and N is the total number of trials in both categories. Note that the z-test assumes that the data are independently sampled from a normal distribution, which was proved with both the Chi-square goodness-of-fit test (Matlab function chi2 g of.m) and the Lilliefors test (Matlab function lillietest.m; [70]).The significance of z is determined according to the critical values from a standard normal distribution. This was done by computing the probability density function of the normal distribution with mean 0 and standard deviation 1 at the resulting z-values. A false discovery rate (FDR) correction [71] for correcting multiple comparisons (20 features, 4 classification methods, and 36 participants) was applied to the critical alpha level of .05. This resulted in a critical alpha level of $p \leq .0086$.

In addition, the effect size of this improvement can be measured directly with the improvement over chance criterion (IOCC):

$$IOCC = \frac{H_0/N - HC/N}{1 - HC/N} \tag{3}$$

For each group and each feature we reported the average p-values and average IOCC-values. To compare the classification methods (discriminant analysis, knn classification with k = 1 and k = 3, SVM), we compared the number of significantly above-chance accuracies for C_{prop} of the features separately in each participant group and separately for the numbers with and without FDR-correction. Normal distribution of these numbers was evaluated with the Kolmogorov-Smirnov test. Since the numbers were not distributed normally in any of the result-sets of the 4 classification methods and 3 participant groups, a non-parametric Wilcoxon signed rank test was used for the comparisons. The resulting p-values were interpreted at the FDR-corrected threshold of significance. For interpretation of significant results, we calculated also the mean accuracy over all features for each classification method result set (i.e., the mean over the 20 features for each classification method). The feature comparisons were then carried out for the best classification method, only.

We calculated the mean, standard deviation (SD), and the range (maximum-minimum) of each feature, separately for each group.

To find out which features had significantly higher average accuracy, lower SD, and lower range of accuracies than the other features, i.e., which features were significantly better than all other features, we calculated one-sample Wilcoxon signed rank tests for each feature vs. all other features on average accuracy, SD, and range. These 180 results were interpreted at the FDR-corrected threshold of significance $p \leq .0242$.

The same procedure was applied to the number of participants showing above-chance accuracy. That is, we evaluated separately for the C_{prop} results with and without FDR-correction and separately for the 3 participant groups which features yielded significantly more above-chance accuracies than the other features. The level of significance was FDR corrected for these 120 comparisons and resulted in $p \leq .0069$.

Preparation of figures

For graphical purposes, we calculated the group-average and group-standard deviation of the difference in power spectra

between the resting and imagery condition. These values were plotted for each electrode position of a subset of 13 electrodes. This subset was chosen to ensure that the graphics are not overloaded.

In addition, a graphical representation of coherences was prepared. We evaluated the difference between rest and imagery in the coherences statistically. This was done by first calculating the coherence over all trials instead of individually for each trial and then converting each coefficient for imagery and rest of each channel × channel combination and each frequency into a z-score using Fisher's r-to-z transformation. Then, taking into account the number of samples and trials for each condition (imagery and rest), these z-scores were compared using formula 2.8.5 from Cohen and Cohen [72].

Then, we calculated the number of subjects with significantly different coherences with a threshold of the FDR-corrected level of significance $p < .0036$ for a subset of 13 electrodes (the same subset as for power spectra). The graphics were constructed by ordering the electrode positions in a scalp-like manner for each frequency and by coding the numbers of participants with significantly different coherences with line-thickness and coloring.

Acknowledgments

Thanks to Joe Miller and Heili Jutglar for language editing. Thanks to Johannes Klackl for help with EEG-recordings. No conflicting financial interests or commercial considerations are related to the presented issues.

Author Contributions

Conceived and designed the experiments: YH JB MK. Performed the experiments: YH JB JC ES. Analyzed the data: YH AT KB. Contributed reagents/materials/analysis tools: YH PH. Wrote the paper: YH JC RN ET.

References

1. Bekinschtein T, Coleman M, Niklison J, Pickard J, Manes F (2009) Can electromyography objectively detect voluntary movement in disorders of consciousness? J Neurol Neurosurg Psychiatry 79: 826–838.
2. Boly M, Coleman M, Davis M, Hampshire A, Bor D, et al. (2007) When thoughts become action: An fMRI paradigm to study volitional brain activity in non-communicative brain injured patients. Neuroimage 36: 979–992.
3. Cruse D, Chennu S, Chatelle C, Bekinschtein T, Fernandez-Espejo D, et al. (2011) Bedside detection of awareness in the vegetative state: a cohort study. Lancet 378: 2088–2094.
4. Fellinger R, Klimesch W, Schnakers C, Perrin F, Freunberger R, et al. (2011) Cognitive processes in disorders of consciousness as revealed by eeg time-frequency analyses. Clin Neurophysiol 122: 2177–2184.
5. Goldfine A, Victor J, Conte M, Bardin J, Schiff N (2011) Determination of awareness in patients with severe brain injury using EEG power spectral analysis. Clin Neurophysiol 11: 2157–2168.
6. John E, Halper J, Lowe R, Merkin H, Defina P, et al. (2011) Source imaging of QEEG as a method to detect awareness in a person in vegetative state. Brain Inj 25: 426–32.
7. Lechinger J, Chwala-Schlegel N, Fellinger R, Donis J, Michitsch G, et al. (2012) Mirroring of a simple motor behavior in disorders of consciousness. Clin Neurophysiol 124: 27–34.
8. Liang X, Kuhlmann L, Johnston L, Grayden D, Vogrin S, et al. (2012). Extending communication for patients with disorders of consciousness. J Neuroimaging. 2012 Aug 28. doi: 10.1111/j.1552-6569.2012.00744.x.
9. Monti M, Coleman M, Owen A (2009) Executive functions in the absence of behavior: functional imaging of the minimally conscious state. Prog Brain Res 177: 249–260.
10. Monti M, Vanhaudenhuyse A, Coleman M, Boly M, Pickard J, et al. (2010) Willful modulation of brain activity in disorders of consciousness. N Engl J Med 362: 579–589.
11. Owen A, Coleman M, Boly M, Davis M, Laureys S, et al. (2006) Detecting awareness in the vegetative state. Science 313: 1402–1402.
12. Schnakers C, Perrin F, Schabus M, Majerus S, Ledoux D, et al. (2008) Voluntary brain processing in disorders of consciousness. Neurology 71: 1614–1620.
13. Schnakers C, Perrin F, Schabus M, Hustinx R, Majerus S, et al. (2009) Detecting consciousness in a total locked-in syndrome: an active event-related paradigm. Neurocase 15: 271–277.
14. Goldfine A, Bardin J, Noirhomme Q, Fins J, Schiff N, et al. (2013) Reanalysis of "Bedside detection of awareness in the vegetative state: a cohort study". Lancet 381: 289–291.
15. Höller Y, Bergmann J, Kronbichler M, Crone J, Schmid E, et al. (2012) Real movement vs. motor imagery in healthy subjects. Int J Psychophysiol 87: 35–41.
16. Schnakers C, Vanhaudenhuyse A, Giacino J, Ventura M, Boly M, et al. (2009) Diagnostic accuracy of the vegetative and minimally conscious state: Cinical consesus versus standardized neurobehavioral assessment. BMC Neurology 9: 35.
17. Vidaurre C, Schlögl A (2008) Comparison of adaptive features with linear discriminant classifier for brain computer interfaces. Conf Proc IEEE Eng Med Biol Soc 2008: 173–176.
18. Bassani T, Nievola J (2010) Brain-computer interface using wavelet transformation and naive bayes classifier. Adv Exp Med Biol 657: 147–165.
19. Sacchet M, Mellinger J, Sitaram R, Braun C, Birbaumer N, et al. (2012) Volitional control of neuromagnetic coherence. Front Neurosci 6: 189.
20. Shoker L, Sanei S, Sumich A (2005) Distinguishing between left and right finger movement from EEG using SVM. Conf Proc IEEE Eng Med Biol Soc 5: 5420–5423.
21. Zhang H, Chavarriaga R, Goel M, Gheorghe L, Millán Jdel R (2012) Improved recognition of error related potentials through the use of brain connectivity features. Conf Proc IEEE Eng Med Biol Soc 2012: 6740–6743.
22. Wang S, James C (2007). Extracting rhythmic brain activity for brain-computer interfacing through constrained independent component analysis. Comput Intell Neurosci, Article ID 41468.
23. Diez P, Mut V, Laciar E, Torres A, Avila E (2009) Application of the empirical mode decomposition to the extraction of features from EEG signals for mental task classification. Conf Proc IEEE Eng Med Biol Soc 2009: 2579–2582.
24. Mohamed A, Marwala T, John L (2011) Single-trial EEG discrimination between wrist and finger movement imagery and execution in a sensorimotor BCI. Conf Proc IEEE Eng Med Biol Soc 2011: 6289–6293.
25. Dauwels J, Vialatte F, Musha T, Cichocki A (2010) A comparative study of synchrony measures for the early diagnosis of alzheimer's disease based on EEG. Neuroimage 49: 668–693.
26. Aloise F, Schettini F, Aricó P, Salinari S, Babiloni F, et al. (2012) A comparison of classification techniques for a gaze-independent P300-based brain-computer interface. J Neural Eng 4: 045012.
27. Boostani R, Graimann B, Moradi M, Pfurtscheller G (2007) A comparison approach toward finding the best feature and classifier in cue-based BCI. Med Biol Eng Comput 45: 403–412.
28. Manyakov N, Chumerin N, Combaz A, Van Hulle M (2011) Comparison of classification methods for p300 brain-computer interface on disabled subjects. Comput Intell Neurosci 2011: 519868.
29. Dias N, Kamrunnahar M, Mendes P, Schiff S, Correia J (2007) Comparison of EEG pattern classification methods for brain-computer interfaces. Conf Proc IEEE Eng Med Biol Soc 2007: 2540–2543.
30. Garrett D, Peterson D, Anderson C, Thaut M (2003) Comparison of linear, nonlinear, and feature selection methods for eeg signal classification. IEEE Trans Neural Syst Rehabil Eng 11: 141–144.
31. Schlögl A, Lee F, Bischof H, Pfurtscheller G (2005) Characterization of four-class motor imagery eeg data for the bci-competition 2005. J Neural Eng 2: L14–22.
32. Nicolas-Alonso L, Gomez-Gil J (2012) Brain computer interfaces, a review. Sensors 12: 1211–1279.
33. Nam C, Woo J, Bahn S (2012) Severe motor disability affects functional cortical integration in the context of brain-computer interface (BCI) use. Ergonomics 55: 581–591.
34. Krusienski D, McFarland D, Wolpaw J (2012) Value of amplitude, phase, and coherence features for a sensorimotor rhythm-based brain-computer interface. Brain Res Bull 87: 130–134.
35. Blokland Y, Vlek R, Karaman B, Özin F, Thijssen D, et al. (2012) Detection of event-related desynchronization during attempted and imagined movements in tetraplegics for brain switch control. Conf Proc IEEE Eng Med Biol Soc 2012: 3967–3969.
36. Höller Y, Bergmann J, Kronbichler M, Crone J, Schmid E, et al. (2011) Preserved oscillatory response but lack of mismatch negativity in patients with disorders of consciousness. Clin Neurophysiol 122: 1744–54.
37. Damborská A, Brázdil M, Rektor I, Janousová E, Chládek J, et al. (2012) Late divergence of target and nontarget ERPs in a visual oddball task. Physiol Res 61: 307–318.
38. Höller Y, Kronbichler M, Bergmann J, Crone J, Schmid E, et al. (2011) Inter-individual variability of oscillatory responses to subject's own name. A single subject analysis. Int J Psychophysiol 80: 227–35.
39. Kotchoubey B, Lang S, Herb E, Maurer P, Birbaumer N (2004) Reliability of brain responses to a person's own name in healthy subjects and patients with brain damage. In: Moore N, Arikan M, editors, Brainwaves and Mind: Recent Advances, Kiellberg, New York. pp. 75–80.

40. Höller Y, Thomschewski A, Schmid E, Höller P, Crone J, et al. (2012) Individual brain-frequency responses to self-selected music. Int J Psychophysiol 86: 206–213.
41. Pfurtscheller G, Brunner C, Schlögl A, Lopes da Silva F (2006) Mu rhythm (de)synchronization and EEG single-trial classification of different motor imagery tasks. Neuroimage 31: 153–159.
42. Abdollahi F, Motie-Nasrabadi A (2006) Combination of frequency bands in eeg for feature reduction in mental task classification. Conf Proc IEEE Eng Med Biol Soc 1: 1146–1149.
43. Hung C, Lee P, Wu Y, Chen L, Yeh T, et al. (2005) Recognition of motor imagery electroencephalography using independent component analysis and machine classifiers. Ann Biomed Eng 33: 1053–1070.
44. Cabrera A, Farina D, Dremstrup K (2010) Comparison of feature selection and classification methods for a brain-computer interface driven by non-motor imagery. Med Biol Eng Comput 48: 123–132.
45. Giacino J, Kalmar K, Whyte J (2004) The JFK coma recovery scale-revised: measurement characteristics and diagnostic utility. Arch Phys Med Rehabil 85: 2020–2029.
46. Coleman M, Rodd J, Davis M, Johnsrude I, Menon D, et al. (2007) Do vegetative patients retain aspects of language comprehension? Evidence from fMRI. Brain 130: 2494–2507.
47. Crone J, Ladurner G, Höller Y, Golaszewski S, Trinka E, et al. (2011) Deactivation of the default mode network as a marker of impaired consciousness: An fMRI study. Plos One 6: e26373.
48. Makeig S, Bell A, Jung T, Sejnowski T (1996) Independent component analysis of electroencephalographic data. Advances in Neural Information Processing Systems 8: 145–151.
49. Jung T, Humphries C, Lee T, Makeig S, McKeown M, et al. (1998) Extended ICA removes artifacts from electroencephalographic recordings. In: Jordan M, Kearns M, Solla S, editors, Climate System Modeling, MIT Press: Cambridge. pp. 894–900.
50. Jung T, Makeig S, Humphries C, Lee T, McKeown M, et al. (2000) Removing electroencephalographic artifacts by blind source separation. Psychophysiology 37: 163–178.
51. McFarland D, McCane L, David S, Wolpaw J (1997) Spatial filter selection for EEG-based communication. Electroencephalogr Clin Neurophysiol 103: 386–394.
52. Schlögl A, Brunner C (2008) BioSig: A free and open source software library for BCI research. Computer 41: 44–50.
53. Hjorth B (1970) EEG analysis based on time domain properties. Electroencephalogr Clin Neurophysiol 29: 306–310.
54. Hjorth B (1975) Time domain descriptors and their relation to particulare model for generation of EEG activity. In: Dolce G, Kunkel H, editors, CEAN Computerized EEG Analysis, Gustav Fischer. pp. 3–8.
55. Pop-Jordanova N, Pop-Jordanova J (2005) Spectrum-weighted EEG frequency ("brainrate") as a quantitative indicator of arousal. Contributions, Sec Biol Med Sci, MASA, XXVI 2: 35–42.
56. Wackermann J (1999) Towards a quantitative characterization of functional states of the brain: from the non-linear methodology to the global linear descriptor. Int J Psychophysiol 34: 65–80.
57. Yang B (2005) Projection approximation subspace tracking. IEEE Trans on Signal processing 43: 95–107.
58. Hurst H (1951) Long-term storage capacity of reservoirs. Trans Am Soc Civ Eng 116: 770–808.
59. Granger C (1969) Investigating causal relations by econometric models and cross-spectral methods. Econometrica 37: 424–438.
60. Ding M, Chen Y, Bressler S (2006) Granger causality: Basic theory and application to neuroscience. In: Schelter S, Winterhalder M, Timmer J, editors, Handbook of Time Series Analysis, Weinheim: Wiley. pp. 438–460.
61. Seth A (2010) A MATLAB toolbox for granger causal connectivity analysis. J Neurosci Meth 186: 262–273.
62. Baccalá L, Sameshima K (2001) Partial directed coherence: a new concept in neural structure determination. Biol Cybern 84: 463–474.
63. Kaminskí M, Blinowska K (1991) A new method of the description of the information ow in the brain structures. Biol Cybern 65: 203–210.
64. Omidvarnia A, Mesbah M, O′Toole J, Coldlitz P, Boashash B (2011) Analysis of the time-varying cortical neural connectivity in the newborn EEG: A time-frequency approach. International Workshop on Signal Processing and their applications (WOSSPA) 7: 179–182.
65. Neumaier A, Schneider T (2001) Estimation of parameters and eigenmodes of multivariate autoregressive models. ACM Trans Math Softw 27: 27–57.
66. Schneider T, Neumaier A (2001) Algorithm 808: Arfit - a matlab package for the estimation of parameters and eigenmodes of multivariate autoregressive models. ACM Trans Math Softw 27: 58–65.
67. Szabó S, Póczos B, Lórincz A (2007) Undercomplete blind subspace deconvolution. journal of machine learning research. J Mach Learn Res 8: 1063–1095.
68. Szabó S, Póczos B, Lórincz A (2012) Separation theorem for independent subspace analysis and its consequences. Pattern Recogn 45: 1782–1791.
69. Marcoulides G, Hershberger S (1974) Statistical Methods: A first course. Psychology Press.
70. Lilliefors H (1967) On the KolmogorovSmirnov test for normality with mean and variance unknown. J Amer Statist Assoc 62: 399–402.
71. Benjamini Y, Hochberg Y (1995) Controlling the false discovery rate: a practical and powerful approach to multiple testing. J R Stat Soc Series B 57: 289–300.
72. Cohen J, Cohen P (1983) Applied multiple regression/correlation analysis for the behavioral sciences. Hillsdale, NJ: Erlbaum.
